# SGLT2 Inhibitors and Their Antiarrhythmic Properties

**DOI:** 10.3390/ijms23031678

**Published:** 2022-01-31

**Authors:** Ewald Kolesnik, Daniel Scherr, Ursula Rohrer, Martin Benedikt, Martin Manninger, Harald Sourij, Dirk von Lewinski

**Affiliations:** 1 Department of Cardiology, University Heart Centre Graz, Medical University of Graz, Auenbruggerplatz 15, 8036 Graz, Austria; ewald.kolesnik@medunigraz.at (E.K.); u.rohrer@medunigraz.at (U.R.); Martin.Benedikt@uniklinikum.kages.at (M.B.); martin.manninger-wuenscher@medunigraz.at (M.M.); dirk.von-lewinski@medunigraz.at (D.v.L.); 2 Department of Endocrinology and Diabetology, Medical University of Graz, Auenbruggerplatz 15, 8036 Graz, Austria; ha.sourij@medunigraz.at

**Keywords:** SGLT2 inhibitors, atrial fibrillation, arrhythmias, ventricular arrhythmias

## Abstract

Sodium-glucose cotransporter 2 (SGLT2) inhibitors are gaining ground as standard therapy for heart failure with a class-I recommendation in the recently updated heart failure guidelines from the European Society of Cardiology. Different gliflozins have shown impressive beneficial effects in patients with and without diabetes mellitus type 2, especially in reducing the rates for hospitalization for heart failure, yet little is known on their antiarrhythmic properties. Atrial and ventricular arrhythmias were reported by clinical outcome trials with SGLT2 inhibitors as adverse events, and SGLT2 inhibitors seemed to reduce the rate of arrhythmias compared to placebo treatment in those trials. Mechanistical links are mainly unrevealed, since hardly any experiments investigated their impact on arrhythmias. Prospective trials are currently ongoing, but no results have been published so far. Arrhythmias are common in the heart failure population, therefore the understanding of possible interactions with SGLT2 inhibitors is crucial. This review summarizes evidence from clinical data as well as the sparse experimental data of SGLT2 inhibitors and their effects on arrhythmias.

## 1. Introduction

**Figure 1:**
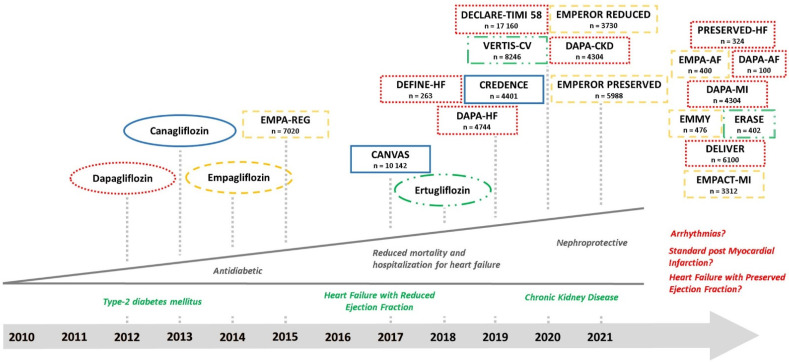
History, completed, and ongoing clinical trials of the SGLT2 inhibitors dapagliflozin (red and dotted), empagliflozin (yellow and dashed), canagliflozin (blue and uninterrupted), and ertugliflozin (green and dashed + dotted). The names of the drugs indicate their approvals from the European Medicines Agency (EMA) or Food and Drug Administration (FDA). Underneath the names of the clinical trials, the number of recruited patients is given.

Sodium-glucose cotransporter 2 (SGLT2) inhibitors are rapidly gaining ground in the treatment of heart failure with reduced ejection fraction (HFrEF). With the first approval for dapagliflozin in the European Union as an antidiabetic drug in the year 2012, multiple clinical trials have been performed with other “gliflozins”. Their indication was already expanded towards heart failure and they are recommended as add-on therapy to RAAS-inhibitors and betablockers in patients with New York Heart Association (NYHA) grades YHA II-IV (at least dyspnea at a level of exertion) in the current update of the American Council of Cardiologists (ACC) consensus decision pathway [1]. More recently, SGLT2 inhibitors were implemented in the heart failure guidelines of the European Society of Cardiology (ESC) for patients with HFrEF [2]. This recommendation is independent of the presence of diabetes mellitus despite the history of this drug class as an anti-diabetic medication. Further, this reflects the ongoing reconceptualization of the molecular mechanisms of SGLT2 inhibitors beyond their glucosuric effects [3]. This paradigm shift is due to the impressive reduction of reported events of hospitalization for heart failure in the respective outcome trials of empagliflozin [4], dapagliflozin [5], canagliflozin [6], sotagliflozin [7], and ertugliflozin [8] in patients with diabetes. Newer data derived from dedicated heart failure trials indicated unaltered benefits in patients with and without diabetes with reduced ejection fraction for dapagliflozin [9], with an ongoing trial in patients with preserved ejection fraction [10]. The same is true for empagliflozin in patients with reduced [11] and preserved ejection fraction [12]. Meta-analyses depict a significant reduction in mortality and an even greater reduction in the number of heart failure hospitalizations [13,14]. Recent trials also suggest nephroprotective effects [15,16] that are reflected by the approval of dapagliflozin in chronic kidney disease independent of diabetes status by the European Medicines Agency in August 2021. The role of SGLT2 inhibitors in the setting of acute myocardial infarction [17] is currently investigated. Figure 1 gives an overview on already finished trials and summarizes the results and ever-expanding indications for SGLT2 inhibitors.

Multiple reasons are discussed as targets for an interaction between SGLT2 inhibitors and myocardium that involve beneficial metabolic effects, such as the upregulation of ketone body, free fatty acid, and branched-chain amino acid utilization [18,19,20], the upregulation of various pathways that counteract detrimental cellular pathways induced by myocardial damage [21,22,23,24,25], anti-inflammatory effects [26], modification of the cellular calcium homeostasis [27], and the modulation of sympathetic influences on the heart. Moreover, an analysis of the available data from the clinical trials in 2016 [28] revealed antihypertensive effects most likely via diuretic/natriuretic activity, and a weight reduction after SGLT2 inhibitor treatment as well. This pattern is confirmed by a recent meta-analysis comparing SGLT2 inhibitors and DPP4 inhibitors [29]. This is of importance since elevated blood pressure is a known risk factor for the development of congestive heart failure [30].

## 2. Arrhythmias and Anti-Arrhythmic Drugs

A major problem of heart failure is the close association to arrhythmias [31]. Underlying mechanisms in the failing heart involve electrolyte disturbances that lead to early and delayed afterdepolarizations because of calcium overload of the myocyte and a prolongation of the action potential duration, electrical automaticity, unidirectional blocks, and re-entry. An always aggravating dysfunction in the neurohumoral balance and chronic stretch of the dilated ventricle favor the occurrence of arrhythmias [32]. A common terminal event of heart failure is sudden cardiac death (SCD) due to an arrhythmogenic event. Excitation–contraction coupling and its synchronized conduction within the heart is essential to prevent arrhythmias.

Many substance groups have been proven in the treatment and prevention of ventricular and supraventricular arrhythmias. Their mechanism of action directly targets the conduction system of the heart. The best ones to show a significant benefit were class-I (sodium-channel-blockers), class-II (beta-blockers), class-III (potassium-channel-blockers), and class-IV (calcium-channel-blockers) anti-arrhythmic drugs, and they are commonly used in daily routines in the prevention and treatment of supraventricular and ventricular arrhythmias as well as in heart failure, according to the ESC Guidelines.

Sodium channels are located in different types of tissues and play a central role in the regulation of membrane potentials. They are highly expressed on cardiomyocytes and induce the initial depolarization of action potentials. Based on this effect, sodium-channel-blockers suppress the fast sodium inward current and stabilize the membrane potential by preventing the formation of action potentials. Therefore, these agents are recommended in the acute and chronic setting of atrial fibrillation [33] and ventricular tachycardia [34]. In the early 1990s, class-I antiarrhythmic drugs were administrated to patients after a myocardial infarction during the Cardiac Arrhythmia Suppression Trials (CAST). Hopes of avoiding SCD events by preventive use of class-I antiarrhythmic drugs were soon crushed, as these drugs showed an excess in mortality due to shock and arrhythmias after myocardial infarction [35,36]. Similar results were observed in patients with established cardiac diseases and complex arrhythmias [37]. Therefore, class-I antiarrhythmic drugs are not recommended for the treatment of arrhythmias in the presence of acute and chronic heart failure [2], and the therapy in this setting is limited to other drugs as preventive or therapeutic measures.

The sympathetic nervous system and its receptors play a central role in the regulations of the heart rhythm by activating neuro-humoral mechanisms in response to stress, fear and physical exercise. β-adrenergic receptors on the cardiomyocytes complex may activate complex intracellular signal cascades that regulate the heart rate by directly binding to the hyperpolarization-activated cyclic nucleotide-gated cation (HCN-pacemaker) channels on nodal cells, resulting in a shortening of the conducting time. Furthermore, β-adrenergic receptors augment the cardiac output by increasing the amount of intracellular Ca^2^^+^ as well as concomitantly decreasing the myofilament Ca^2^^+^ sensitivity [38,39]. The potential pro-arrhythmic effects of β-adrenergic action is elevated with disturbed ion concentrations in the blood, nerve remodeling [40], hypertrophy, and fibrosis [41]. Based on these molecular findings, β1-selective beta-blockers are the treatment of choice in the prevention of supraventricular and ventricular tachycardia by suppressing sympathetically-mediated triggers, functional re-entrant substrates, and slowing of the sinuatrial and atrioventricular nodal rates, according to the recent guidelines. Additionally, β1-selective beta-blockers form the baseline therapy in patients with chronic HFrEF next to angiotensine converting enzyme (ACE) inhibitors or angiotensin II receptor blockers (ARB), as well as SGTL2-inhibitors and mineral receptor antagonists (MRA) due to their anti-remodeling effect.

Potassium channels are expressed like sodium channels in various tissues. Potassium channels induce mainly a fast potassium outward current resulting in hyperpolarization and termination of the action potential. Potassium-channel-blockers such as amiodarone prolong the action potential via an inhibited potassium outflow. As more potassium stays inside the cardiomyocyte for a longer period, amiodarone exerts a negative bathmothropic effect by stabilizing the membrane potential and preventing the formation of ectopic arrhythmias. This mechanism is used in the treatment of supraventricular and ventricular arrhythmias. However, amiodarone may exert significant side effects through its accumulation in different organ systems such as the lung, eyes, liver, skin, and nervous system. Furthermore, the drug consists of iodine that may interact with the thyroid gland. Therefore, the recommendation for an amiodarone therapy is limited to situations where other antiarrhythmic drugs are contraindicated. However, routine use of amiodarone in patients with congestive heart failure and ventricular arrhythmias did not result in a significant reduction of mortality [42,43,44], and is inferior compared to an implanted cardioverter-defibrillator only strategy [45].

Non-dihydropyridine calcium channel blockers exert their effect by directly targeting the L-type calcium channel. A lower calcium influx leads to a negative chronotropic, a negative inotropic, and a negative dromotropic effect. These drugs are also recommended for the treatment of supraventricular tachycardia on a similar level as beta-blockers. However, due to early alarming data of increased mortality in the setting of HFrEF [46,47], neutral effects compared to placebo [48,49], and no available data on significant reduction of mortality in HFrEF, these drugs play a pivotal role in the combined management of arrhythmia and heart failure.

All mentioned antiarrhythmic drugs are under investigation and have been in clinical use for decades, while SGLT2 inhibitors are a relatively new player in this field. To understand potential antiarrhythmic effects of SGLT2 inhibitors, one must look beyond a direct interaction with the electrical conduction system of the heart. However, antiarrhythmic properties have not been prospectively investigated for SGLT2 inhibitors so far. In general, little is known on antiarrhythmic properties of SGLT2 inhibitors.

## 3. Heart Failure and Diabetes: Ventricular Arrhythmias

**Figure 2:**
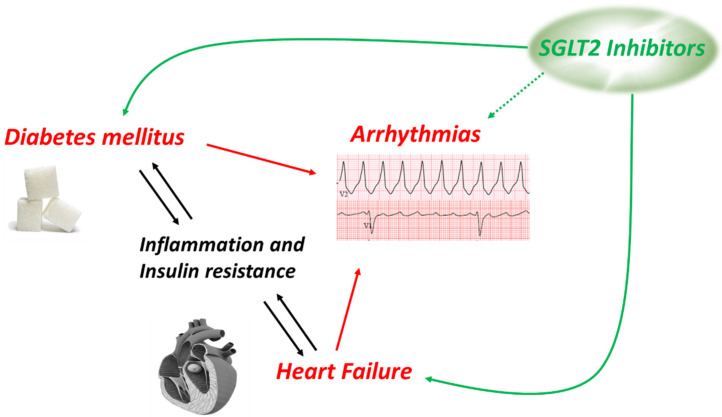
Connection and interaction between diabetes mellitus, heart failure, and arrhythmias.

Contractility of the heart is a very energy demanding process, and the heart is therefore enabled to use various energetic substrates as a so-called “metabolic omnivore”. However, throughout the development and in various pathologies, preferences of substrate utilization are changed or regulated [50]. Healthy adult hearts use fatty acids and carbohydrates as their predominant fuel, but cardiac disease such as hypertrophy and heart failure often lead to a more prominent use of glycolytic energy production [51]. In the presence of diabetes on the other hand, the myocardium relies more on the metabolism of fatty acids, which is not as efficient as a mixed metabolism consistent with the utilization of both fatty acids and glucose. Following the increased consumption of fatty acids, their metabolites such as diacylglycerol tend to accumulate in the myocardium [52]. This leads to increased interstitial and perivascular fibrosis, a histological finding that defined the term “diabetic cardiomyopathy” in the early 1970s [53]. Therefore, it is not surprising that the incidence rates for heart failure are about twice as high in patients with than without diabetes [54]. On the other hand, the prevalence of diabetes is high in heart failure as well [55]. As shown in Figure 2, heart failure and diabetes mellitus interact bi-directionally mainly via inflammatory signaling [56,57] and insulin resistance [58,59], and both diseases are risk factors for arrhythmias on the ventricular and on the atrial level. A disturbed metabolic pattern alone is discussed to promote arrhythmias and SCD [60,61] and may be a point of action for SGLT2 inhibitors, that demonstrated to stabilize an impaired state of energy consumption of the heart [18]. In addition to some data in animal (mainly rodent) models, very little data are generated from human tissue due to the limited availability. Therefore, using human-induced pluripotent stem cells was an innovative approach [62]. High glucose treatment induces a cellular hypertrophy, reduced contractility, and changes of the expression levels of the ryanodine receptor and the sodium-calcium exchanger (NCX). Empagliflozin ameliorates high glucose-induced cardiac dysfunction on all mentioned levels. This intriguing data, however, must be interpreted with caution as the authors also describe a robust expression of SGLT2 in their cells which is markedly upregulated in their disease model. However, expression of SGLT2 has neither been detected in human atrial [63] nor ventricular [64] myocardium.

Diabetes is an independent risk factor for SCD as well [65]. Data of the large ARIC Study with a follow-up period of 12 years revealed a 2.6-fold increase in patients with manifest diabetes [66]. Heart failure and diabetes interact bi-directionally mainly via inflammation and insulin resistance [67]. SGLT2 inhibitors combine beneficial effects in the conditions of heart failure with robust antidiabetic properties. Therefore, antiarrhythmic effects can be suspected and have already been demonstrated in animal models. In a rat model, empagliflozin treatment significantly ameliorates sotalol-induced prolongation of the QTc interval [68]. In an ex vivo model of global ischemia-reperfusion, empagliflozin reduced ventricular arrhythmia vulnerability in rabbit hearts via SGLT2-independent mechanisms [69]. In line with these findings, an in vivo experimental series in male Sprague Dawley rats showed that empagliflozin pretreatment could completely avoid the occurrence of lethal ventricular arrhythmias after ligation of the left main coronary artery for five minutes followed by a reperfusion of 20 min. In the control group, 69% of all rats died due to ventricular tachycardia. An inhibitor of the ERK1/2 pathway abolished the effect of empagliflozin, making this the pathway a potential downstream target [70]. A reduced burden of ventricular arrhythmias following ischemia-reperfusion could also be observed in rats treated with dapagliflozin [24]. Dapagliflozin was also demonstrated to suppress prolonged ventricular repolarization in rats with metabolic syndrome induced by a high carbohydrate diet [71]. Of note, these animals were all non-diabetic. At least empagliflozin seems to target the epicardial fat tissue as well. Adipocytes located in the epicardial region are able to secrete adipokines that exert effects on the expression levels of ion channels in cardiomyocytes. In mice with metabolic syndrome, adipokines induced a decrease in the expression level of potassium channels and an increase in the expression levels of calcium channels. Empagliflozin pretreatment could attenuate this effect in mice with metabolic syndrome, potentially reducing the risk of arrhythmias due to a disturbed ion homeostasis [72]. For the other SGLT2 inhibitors canagliflozin and ertugliflozin, as well as for the combined SGLT1/SGLT2 inhibitor sotagliflozin, no data regarding antiarrhythmic effects have been published so far.

## 4. SGLT2 Inhibitors and Ventricular Arrhythmias: Evidence from Clinical Trials

A recent meta-analysis that analyzed all published clinical trials with SGLT2 inhibitors until December 2020, including 68 trials with a total number of 63,166 patients of which 35,883 (56.8%) received an SGLT2 inhibitor, found a reduced rate of SCD events in their intervention groups [73] with a relative risk reduction of 28%. Attention must be paid to the fact that the term “SCD” consists of “sudden cardiac death”, “sudden death”, and “cardiac arrest”. Results were only significant for the “sudden cardiac death” component. There was no significant difference in the occurrence of ventricular arrhythmias between the SGLT2 and the placebo group. As a limitation, not all trials reported these events and the overall incidence rate was very low, with only 220 events of ventricular arrhythmias in 49,963 patients (=0.4%) and 187 events of “sudden cardiac death” in 45,483 patients (=0.4%). Nevertheless, for other established heart failure drugs a reduced burden of ventricular arrhythmias could be demonstrated. A recent study analyzed 151 patients with HFrEF and implanted cardioverter defibrillators (ICD) who were switched from ACE inhibitors or ARB to valsartan/sacubitril. Within one year of observation the burden of ventricular arrhythmias expressed by ICD interventions dropped significantly [74]. This effect for sacubitril/valsartan could also be extrapolated from the original PARADIGM-HF trial [75]. Another established heart failure therapy that reduces ventricular arrhythmias are beta-blockers [76]. The same is true for MRA, however, ACEi and ARB alone failed to reduce the incidence of ventricular arrhythmias and SCD [77]. The EMBODY trial prospectively enrolled patients with diabetes after acute myocardial infarction and empagliflozin-treatment improved parameters, reflecting sympathetic and parasympathetic nerve activities that were measured mainly via Holter ECG monitoring [78]. Another trial investigated acute effects of dapagliflozin in 19 patients with type 2 diabetes within a period of two weeks. Here, a reduced ventricular ectopic burden suggests an early antiarrhythmic benefit induced by dapagliflozin [79]. A post hoc analysis from the recently published dapagliflozin in patients with HFrEF (DAPA-HF) trial provides the first strong evidence for a clinical benefit in the setting of HFrEF. Of the participating 4744 patients, 335 patients (=6.6%) experienced the composite of a serious ventricular arrhythmia, resuscitated cardiac arrest, or sudden death. There were significantly lower events in the dapagliflozin than in the placebo group (5.9% versus 7.4%) with a relative risk reduction of 21%. As a limitation, the authors report a potential under-reporting of events, since ventricular arrhythmias have not been a prespecified trial outcome [80]. Still, until today no larger clinical trial explored the antiarrhythmic properties of SGLT2 inhibitors in a prospective manner. However, this research question will be clarified within the next years since patients with heart failure are often treated with an ICD or cardiac resynchronization therapy (CRT). An ICD can monitor and treat episodes of ventricular tachyarrhythmia while a CRT can only monitor these episodes. Therefore, the EMPA-ICD trial was launched in April 2019 and will investigate the impact of empagliflozin on the burden of ventricular arrhythmias in patients with diabetes and an implanted ICD or cardiac resynchronization therapy (CRT) device [81] (trial number: jRCTs031180120—Japan). Another trial that has been initiated in June 2021 is the ERASE-trial (trial number: NCT04600921), a multi-center phase III study located in Austria that investigates the effect of ertugliflozin on the burden of ventricular arrhythmias in heart failure patients treated with an ICD or CRT irrespective of the diabetes status.

## 5. Heart Failure and Diabetes: Atrial Fibrillation

Similar to the interaction between heart failure and diabetes, both diseases predispose to AF development through both electrical as well as structural remodeling of the atria [82,83]. Indeed, atrial fibrillation is common in heart failure patients and predicts worsened outcomes independent of NYHA class or left ventricular ejection fraction [84], and the risk for new onset atrial fibrillation is increased by approximately 40% in patients with diabetes mellitus [85]. As atrial fibrillation is a major risk factor for ischemic stroke and responsible for approximately 20–30% of all ischemic strokes [33], the calculated 2.27-fold increased risk for cerebral thromboembolism in patients with diabetes indicates a significant clinical problem [86].

Data derived from animal models demonstrate an interaction between SGLT2 inhibitors and atrial myocardium. Mice with induced diabetes were treated with empagliflozin or placebo, and empagliflozin successfully ameliorated atrial structural and electrical remodeling, expressed by reduced left atrial diameter, reduced interstitial fibrosis, and reduced incidence of atrial fibrillation. Broad analysis of potentially involved proteins depicted a PGC-1a/NRF-1/Tfam pathway causing these beneficial effects [87]. Similar results were observed in rats with induced metabolic syndrome that were treated with the combined SGLT1/SGLT2 inhibitor sotagliflozin. Treatment with sotagliflozin counteracted left atrial enlargement in vivo and reduced spontaneous calcium release events in vitro [88]; the latter events are typically observed in conditions of atrial fibrillation [89]. Dapagliflozin treatment has been demonstrated to reduce epicardial fat volume in human patients with diabetes mellitus [90], and according to an analysis from the Framingham Heart Study, epicardial fat volume is directly associated with prevalent atrial fibrillation [91], potentially via adipokines. Distinct clinical data analyzing P-wave indices as a surrogate for conduction velocity within the atria were recently provided in a small trial using dapagliflozin in patients with diabetes [92]. Treatment for 6 months resulted in significantly decreased P-wave dispersion, P-wave variation changes, and epicardial fat volume compared to the control group. As higher values of these P-wave indices are considered to be risk factors for atrial fibrillation, this might highlight a potential mechanistical link. Another point of action currently discussed for SGLT2 inhibitors is their impact on mitochondrial dysfunction in atrial remodeling independent of the presence of diabetes. Mitochondria-protective effects of SGLT2 inhibitors could thus provide benefits in patients with and without diabetes to a similar extent [93].

## 6. SGLT2 Inhibitors and Atrial Arrhythmias: Evidence from Clinical Trials

A recent analysis of the large FDA adverse event reporting system including >700,000 adverse events revealed a lower incidence of atrial fibrillation in diabetic patients treated with SGLT2 inhibitors if compared to other glucose-lowering drugs. This highly significant finding was also consistent after excluding reports on used antiarrhythmic drugs, renal disease and/or other cardiovascular disease, indicating a robust antiarrhythmic effect [94]. Another meta-analysis including all clinical trials that investigated SGLT2 inhibitors until December 2020 also reports a significant reduction of the incidence of atrial arrhythmias with an overall prevalence of approximately 1% of the study population [73]. A pooled analysis of 31 randomized clinical trials including more than 75,000 patients found that SGLT2 inhibitor use is associated with a lower incidence and recurrence of atrial fibrillation as well as with a reduced rate of cardiovascular outcomes [95]. Conversely, these findings could not be retraced in another meta-analysis dealing with a similar yet older database based on reported trial data until October 2019 [96]. A post hoc analysis from the DECLARE-TIMI 58 investigated the incidence of the first episode as well as the total number of reported episodes of atrial arrhythmias in 17,160 patients with type 2 diabetes treated with dapagliflozin or placebo. Dapagliflozin decreased the incidence of atrial arrhythmias with a relative risk reduction of 19%. These effects were independent of a prior known atrial arrhythmia. However, the absolute number of 589 events (= 3.4% in relation to the total study population) over an observation period of 4 years seems pretty low, and the authors acknowledge this limitation [97]. Another trial based on a Taiwanese multi-center healthcare provider reports a lower incidence rate of atrial fibrillation after SGLT2 inhibitor treatment compared to dipeptidyl peptidase 4 (DPP4) inhibitor treatment in more than 25,000 patients. Although these are real-world data, the study design was retrospective and must be interpreted with caution [98]. A major limitation of nearly all clinical trials dealing with antidiabetic medications is the fact that they do not routinely report atrial arrhythmias in their primary analysis. Even regular ECG follow up is usually not routinely performed in trials focusing on antidiabetic drugs. This might have led to a haziness in the reported numbers, as many episodes as well as the potential therapeutic effect of these drugs might have been missed [99]. Within a selected population with diabetes and cardiovascular risk factors or established cardiovascular diseases, one would expect a higher prevalence of atrial fibrillation, since the prevalence of atrial fibrillation is approximately 2.3% in people older than 40 years [100] and the population in the reported clinical trials is far older. Definite conclusions cannot be drawn from available clinical data, and it remains unclear if a potential beneficial SGLT2 inhibitor effect on atrial fibrillation might only be due to the heart failure therapy or if it was the result of direct interaction with the myocardium [101]. Lower rates of atrial fibrillation might be attributed to reduced blood pressure or body weight, as observed in patients with and without diabetes. Interestingly, none of the large SGLT2 inhibitor trials has demonstrated a reduction in the rate of stroke, which appears to be in conflict with the reduction in atrial fibrillation hypothesis. An explanation might be the rather short observation periods in these trials, that do not suffice to observe a potential reduction in atrial fibrillation, which subsequently could translate into reduced stroke rates. Similar to ventricular arrhythmias, no clinical trial has prospectively investigated the impact of SGLT2 inhibitors on atrial fibrillation. At the very least, the ongoing ERASE trial will provide evidence for ertugliflozin and its influence on the burden of atrial arrhythmias in people with heart failure treated with an ICD or CRT. Trials for empagliflozin (EMPA-AF; NCT04583813) and dapagliflozin (DAPA-AF; NCT04792190) will provide data in the setting of atrial fibrillation within the next years.

## 7. Molecular Research of SGLT2 Inhibitors Connected to Arrhythmias

Despite promising data of SGLT2 inhibitors as heart failure drugs with possible antiarrhythmic properties, molecular mechanisms have not been identified yet. A classic cell receptor-based signaling cascade within myocytes seems unlikely because results derived from in vivo and in vitro experiments are too varying, even if the effects could be triggered via other receptors than SGLT2. Given that SGLT2 inhibitors are all small molecules with molecular masses of lower than 500 g/mol, these substances could easily be taken up and metabolized or modified by cardiomyocytes and exert effects inside the cell. Interestingly, no consistent effects could be identified, not even for a single substance. Therefore, big data analysis using deep learning artificial intelligence seems to be a reasonable approach to reveal the most likely targets. An algorithm that analyzed publicly available databases showed that empagliflozin could reverse 59% of all known protein alterations in heart failure with preserved ejection fraction (HFpEF) with a predominance of the effect via the sodium hydrogen antiporter 1 (NHE1) receptor and the impact on oxidative stress modulation, myocardial stiffness, myocardial extracellular matrix remodeling, and systemic inflammation [102]. None such analysis has been performed for another SGLT2 inhibitor so far. However, the complexity of these effects, especially in the presence of myocardial damage, has recently been summarized by the authors and involves upregulations of the JAK/STAT3 [21,22], the ERK 1/2 [70], the cGCH1-BH4/NO [23], the B-cell lymphoma 2 gene [24], and the AMPK [18,25,26] pathways. A modification of adipokines from epicardial fat may play a role as well [72]. Although these mechanisms of action for SGLT2 inhibitors were observed after more or less acute myocardial damage, an improvement of the myocardial function will likely prevent the development of ventricular arrhythmias [103] in the long term. On the atrial level, the current evidence available involves the PGC-1a/NRF-1/Tfam pathway [87] and modifications of the sodium-calcium exchanger (NCX) protein [88] after SGLT2 inhibitor treatment. With respect to all identified and concealed downstream pathways, a multifactorial mechanism of action seems currently the most obvious explanation for the overall beneficial outcomes of clinical trials and a diversity of upregulated proteins after SGLT2 inhibitor treatment.

## 8. Conclusions

SGLT2 inhibitors are fully implemented as heart failure drugs due to their impressive outcomes in clinical trials. Their strong and consistent effect on heart failure hospitalization indicates improved cardiac function and is likely leading to lower numbers of ventricular arrhythmias. On the atrial level, post hoc analysis of large clinical trials revealed relevantly reduced incidence rates of atrial fibrillation. On the ventricular level, at least one post hoc analysis demonstrated a significant reduction of ventricular arrhythmias. We can expect the first evidence from ongoing clinical trials within the next years. Data derived from animal and cellular models support the hypothesis that SGLT2 inhibitors exert anti-arrhythmogenic effects. However, similar to the overwhelming beneficial effects with respect to heart failure, no consistent pathway or mechanism has been identified for the antiarrhythmic properties.

## Data Availability

Not applicable.
